# Draft Genome Sequence of *Microbacterium oleivorans* Strain A9, a Bacterium Isolated from Chernobyl Radionuclide-Contaminated Soil

**DOI:** 10.1128/genomeA.00092-17

**Published:** 2017-04-06

**Authors:** Philippe Ortet, Nicolas Gallois, Laurie Piette, Justine Long, Catherine Berthomieu, Jean Armengaud, Mohamed Barakat, Virginie Chapon

**Affiliations:** a CEA, CNRS, Aix-Marseille Université, UMR 7265 Biologie Végétale et Microbiologie Environnementales, Laboratoire des Interactions Protéine Métal, Saint-Paul-lez-Durance, France; b CEA, CNRS, Aix-Marseille Université, UMR 7265 Biologie Végétale et Microbiologie Environnementales, Laboratoire d'Écologie Microbienne de la Rhizosphère et d'Environnements Extrêmes, Saint-Paul-lez-Durance, France; c CEA, DRF/IBiTec-S/SPI/Li2D, BP 17171, Bagnols-sur-Cèze, France

## Abstract

Here, we present the draft genome sequence of *Microbacterium oleivorans* strain A9, a uranium-tolerant actinobacterium which has been isolated from radionuclide-contaminated soil from the Chernobyl exclusion zone. It is composed of 22 contigs totaling 2,954,335 bp and contains 2,813 coding DNA sequences, one cluster of rRNA genes, and 45 tRNA genes.

## GENOME ANNOUNCEMENT

Members of the genus *Microbacterium* are rod-shaped, Gram-positive, and non-spore-forming actinobacteria. *Microbacterium*-related bacteria are apparently ubiquitous, since they have been detected in a wide variety of habitats, including soil (1), sediment (2), air (3), seawater (4), plants (5), jellyfish (6), insect gut (7), food (8), clinical specimens (9), polluted environments (10), and radionuclide-rich soils (11). At the time of this writing, there are more than 90 *Microbacterium* species with a valid name (12) and 129 complete or draft genomes (https://gold.jgi.doe.gov). Here, we report the draft genome sequence of *Microbacterium oleivorans* strain A9, which was isolated from a radionuclide-contaminated soil sample collected from trench T22 in the Chernobyl exclusion zone (13). This bacterium exhibits high uranium tolerance due to multiple detoxication mechanisms (14).

Bacteria were cultured in LB at 32°C until late-exponential-growth phase, and high-quality genomic DNA was extracted from cells using the DNeasy blood and tissue kit (Qiagen), according to the manufacturer’s instructions for Gram-positive bacteria. The quality of the purified DNA was checked using a NanoDrop spectrophotometer (Thermo Scientific). Genomic DNA was sequenced on a HiSeq 2000 sequencing platform (Illumina) by the GenoScreen Company (Lille, France). *De novo* genome assembly was performed on the reads using ABySS (15).

A total of 18,500,514 reads were obtained, with a mean read length of 100 bases. These reads were assembled into a set of 104 contigs for a total of 2.99 Mbp sequence by the assembler, with k-mer optimal size set to 64. Of those 104 contigs, only the 22 contigs with sequence length longer than 500 bp were retained. The largest contig was 553,625 bp, and the *N*_50_ parameter was 205,808 bp. The genome size was estimated at 2.95 Mbp. According to our assembly, the G+C content was 68.33%, a value in accordance with the phylogenetic position of *Microbacterium* within the *Actinobacteria* phylum (16). Taxonomic assignment at the species level was provided by the NCBI using the average nucleotide identity, as described by Federhen et al. (17). Genome annotation was performed with the NCBI Prokaryotic Genome Annotation Pipeline (18), which reported the prediction of 2,813 protein-coding genes, 15 pseudogenes, one complete rRNA cluster, three noncoding RNAs (ncRNAs), and 45 tRNA genes. Annotation of regulatory proteins was accomplished using the P2RP Web server (19), which identified genes encoding 58 two-component system proteins (30 histidine kinases, 26 response regulators, and two histidine phosphotransferases) and 180 transcription factors.

### Accession number(s).

This whole-genome shotgun project has been deposited at DDBJ/EMBL/GenBank under the accession no. MTIO00000000. The version described in this paper is the first version, MTIO01000000.
